# Aging in place preferences of older adults with cognitive impairment and informal caregivers: A qualitative analysis of a discrete choice experiment

**DOI:** 10.1177/13872877261469138

**Published:** 2026-07-23

**Authors:** Soukaina El Jaouhari, Caroline Geetan, Bachar Farousi, Luis I. Pilli, David Neal, Thomas Engelsma, Isabelle Vullings, Özgül Uysal-Bozkir, Floor Van Deudekom, Majon Muller, Janet Macneil Vroomen

**Affiliations:** 1 Department of Internal Medicine and Geriatrics, 26066 Amsterdam UMC, University of Amsterdam, Amsterdam, The Netherlands; 2 Ageing & Later Life section, Amsterdam Public Health Research Institute, Amsterdam, The Netherlands; 3 Erasmus School of Health Policy & Management, 6984 Erasmus University Rotterdam, Rotterdam, The Netherlands; 4 Erasmus Choice Modelling Centre, 6984 Erasmus University Rotterdam, Rotterdam, The Netherlands; 5 Department of Medical Informatics, eHealth Living & Learning Lab Amsterdam, 26066 Amsterdam UMC, Amsterdam, The Netherlands; 6 Digital Health section, Amsterdam Public Health Research Institute, Amsterdam, The Netherlands; 7 Erasmus School of Social and Behavioral Sciences, Department of Psychology, Education and Child Studies, 6984 Erasmus University Rotterdam, Rotterdam, The Netherlands; 8 Department of Geriatrics, 1204 OLVG, location West, Amsterdam, The Netherlands; 9 Department of Internal Medicine and Geriatrics, 26066 Amsterdam UMC, VU University, Amsterdam, The Netherlands; 10 Amsterdam Cardiovascular Sciences, Amsterdam, The Netherlands

**Keywords:** aging in place, Alzheimer's disease, discrete choice experiments, home care services, informal caregivers, patient preferences, Persons with dementia, think-aloud method

## Abstract

**Background:**

The Netherlands spends more on long-term care than any other country. In 2015, reforms aimed to promote aging in place (AIP) but have been associated with increased crisis-related hospitalizations, rising caregiver burden, and a need for safer AIP strategies. Little is known about how people with dementia and informal caregivers prioritize AIP care and how their preferences differ.

**Objective:**

To identify and compare AIP care preferences of people with dementia and informal caregivers, highlighting shared and individual priorities.

**Methods:**

We used a think-aloud protocol within a discrete choice experiment (DCE). Participants were recruited from outpatient clinics, daycare centers, and cultural organizations. To ensure inclusivity, a formal dementia diagnosis was not required given documented diagnostic delays, particularly in migrant communities. Forty participants (20 care recipients, 20 informal caregivers) completed choice tasks comparing care packages. The DCE comprised three rounds: an online survey for informal caregivers, interviews with the person with dementia, and a dyadic round. Think-aloud responses illuminated decision-making; audio was transcribed verbatim and thematically analyzed.

**Results:**

Five themes shaped preferences: (1) burden of in-home care and care planning, (2) role of case managers, (3) accessibility and inclusivity of social activities, (4) trust in healthcare providers, and (5) reimbursements for home adaptations. Care recipients prioritized in-home care and social activities; caregivers emphasized case management. Both valued emotional support and full reimbursements for home adaptations.

**Conclusions:**

Tailoring AIP strategies to the distinct needs of both groups is essential and can support more equitable, effective AIP models.

## Introduction

Dementia affects not only persons with dementia, but also their families, caregivers and society at large, leading to profound health, social and economic consequences.^1^ Moreover, dementia is among the most costly global health conditions, with annual societal costs estimated at $1.3 trillion annually.^2^ Long-term care (LTC) plays a central role, and persons with dementia constitute up to 80% of the nursing home population.^3^ This high prevalence is a primary driver of the demand for LTC services, as the progressive decline in performing activities of daily living (ADL) necessitates formal support that often exceeds the capacity of informal caregivers. LTC services aim to support individuals to live as independently as possible, either at home or in institutional settings.^4^ In the European Union, the number of people requiring LTC is expected to increase from 30.8 million in 2019 to 38.1 million in 2050.^5^ Furthermore, the global prevalence of dementia continues to rise: while approximately 55 million were affected in 2019, this number is expected to nearly triple to 139 million by 2050.^2^ To address this demand, many European member states have reformed LTC systems, prioritizing aging in place (AIP) over institutional care, which is generally viewed as more cost effective and aligned with preferences of older adults.^6^

The Netherlands spends at least 4.4% of its gross domestic product on LTC services, making it the country with the highest expenditure among the countries of the Organization for Economic Cooperation and Development (OECD).^7,8^ A 2015 reform sought to reduce costs and ensure sustainability by shifting to AIP, emphasizing informal caregiver and community-based care services while safeguarding access and quality.^9^ The reforms was associated with reduced nursing home admissions and prolonged community living for persons with dementia.^10,11^ However these benefits were also accompanied by unintended consequences, including more unplanned hospitalizations and greater caregiver burden, underscoring the need for more effective AIP strategies.^12^

Although prior research has examined care preferences of persons with dementia and informal caregivers, little is known about how these groups differently prioritize care within the context of AIP. Gaining insight into these differences is essential for designing tailored and effective care models.

Previous studies have identified key components of successful AIP, including place, social networks, technology, and personal characteristics such as resilience.^13^ However, the experience of AIP is heavily influenced by the progression of the disease. Dementia typically advances through three stages: an early stage where individuals remain largely independent; a prolonged middle stage requiring increasing support for daily activities; and a severe stage characterized by total care dependency.^1^ Furthermore, the presence of behavioral and psychological symptoms of dementia (BPSD), such as agitation or apathy, significantly increases the complexity of home-based care.^14^ In the United Kingdom, for example, persons with early-stage dementia prioritized social and recreational activities, along with emotional support, whereas informal caregivers prioritized practical and emotional assistance. In the late stages of dementia, informal caregivers expressed the greatest preference for respite care and help with daily living assistance.^15^ Given significant differences between the UK and the Dutch health-care systems, it remains unclear whether these preference patterns are applicable to the Dutch context. Additionally, it is important to consider whether these preference patterns apply to the Dutch population, including underrepresented groups.

Persons with a migration background are often underrepresented in research, despite having a three- to five-fold increased risk of developing dementia compared to native populations.^16,17^ They also encounter greater barriers to diagnosis, lower access to formal care services, and distinct cultural expectations around caregiving.^18–20^ In the Netherlands, the largest migrant groups aged 65 and over are of Surinamese (2.3%), Indonesian (2.2%), Turkish (1.6%), and Moroccan (1.5%) origin.^21^ Ensuring their inclusion is therefore essential for developing care models that reflect the diversity of care needs and preferences within the Dutch population. This study deliberately emphasized the participation of persons with dementia from migrant backgrounds to ensure findings are inclusive and generalizable for the Dutch population.

To better understand the needs of both persons with dementia and their informal caregivers, this Dutch study used culturally inclusive discrete choice experiments (DCE) with a think-aloud protocol to capture and compare care preferences in the context of AIP.^22^ DCEs elicit preferences by asking participants to make a series of hypothetical choices between alternatives that vary across specific attributes, enabling researchers to estimate the relative importance of these attributes (care scenarios).^23^ Their increased use indicates a broader shift toward patient-centered and participatory healthcare, in which individuals are active contributors to its design. This study identifies and contrasts the preferences of persons with dementia and their informal caregivers. We hypothesize that informal caregivers will prioritize care differently than persons with dementia, generating insights that can inform the design of more responsive inclusive care models.

## Methods

### Study design

This study aimed to identify and compare care preferences of persons with dementia and their informal caregivers regarding AIP. This study used a think-aloud method, in which qualitative data was obtained while respondents were conducting choice tasks within a dyadic DCE.^22,24,25^ The development of the study and the protocol is published elsewhere.^22,26–29^ In this study, the DCE did not serve as an endpoint for quantitative analysis but rather as an instrument to elicit reflection and explore participants’ reasoning for prioritizing care components and services. The stages involved in the development of the DCE used in this study are divided into four phases: 1) attribute development, 2) attribute prioritization and development of attribute levels, 3) a pilot DCE and finally, 4) the final DCE.^22,25,27–29^ Attributes and levels were derived from qualitative research with the target population and informed the initial development of attributes and attribute levels.^27–29^ A feasibility study was done to determine best in-practice dyadic DCE design to enable persons with dementia and informal caregivers to participate in health care decision making.^30^ A flow chart detailing this process can be found in Supplemental Figure 1.

The DCE was conducted in three stages: (1) a solo round completed by the informal caregiver independently (online), (2) a solo round with the person with dementia with a researcher assisting by reading and additionally explaining the tasks if necessary and using a think-aloud method, and (3) a joint dyadic interview facilitated by the researcher and using a think-aloud method. The dyadic interview followed the solo interviews. The think-aloud protocol was applied in the individual round of the person with dementia, and in the dyadic DCE with the person with dementia and their informal caregiver together, encouraging participants to verbalize their decision-making processes.^25^ This qualitative interview approach provided more depth and context to the quantitative DCE data, enhancing understanding of the reasoning behind participants’ choices. This approach also holds methodological value, as it allows exploration of nuanced perspectives and decision-making processes that may not be captured through quantitative measures alone. The study adhered to the COREQ guidelines^31^ to ensure adequate reporting of the qualitative data (Supplemental Table 1).

### Participants and sampling

Persons with dementia, mild cognitive impairment and their informal caregivers were recruited for this study. Individuals who experienced challenges with communication or were not able or willing to provide consent, were excluded from participation. A formal dementia diagnosis was not required to ensure inclusivity, given frequent delays in diagnosis, particularly in migrant communities.^18^ We also included individuals with subjective memory problems, as they are often already considering future care needs and advance care planning, despite retaining greater cognitive reserve. To improve equity and generalizability, inclusion of persons with a migration background was prioritized given their higher prevalence of dementia.^16^ Recruitment was conducted through healthcare organizations and outpatient clinics (OLVG, Amsterdam UMC). To ensure inclusion of persons with a migration background, we recruited through an outpatient clinic at OLVG that is specialized in treating persons with a migration background. In addition, we recruited through community centers, mosques, and through culturally sensitive daycare centers. Participants received written and verbal information, with informed consent obtained; verbal consent was recorded when signing was not possible due to difficulty with writing. All participants were reminded of their right to withdraw at any time.

The Medical Ethics Research Committee of the Amsterdam University Medical Centers confirmed that the Medical Research Involving Human Subject Act did not apply to this research project (W23_122 #23.137).

### Setting

Interviews were conducted at participants’ preferred locations to support accessibility. Most took place in participants’ homes due to mobility limitations, while some were conducted at a culturally sensitive daycare facility and one at Amsterdam UMC. In one case an informal caregiver was not able to fill it in independently due to low literacy, so an interviewer was present.

To reduce caregiver influence on individual responses, informal caregivers were asked to leave the room during interviews with persons with dementia. When their presence was requested for emotional comfort or communication support, interviewers used techniques such as managing turn-taking and gently redirecting input, to ensure responses reflected the participant's own views.

Interviews were conducted in Dutch when feasible. When needed, multilingual researchers conducted interviews in participants’ preferred language, in this study Moroccan Arabic (Darija), to ensure cultural and linguistic suitability. Three trained researchers recruited and interviewed all participants (SEJ is a qualitative PhD researcher; BF and CG were medical master students, professionally trained to conduct the interviews). The team's multilingualism and use of culturally adapted materials helped minimize linguistic barriers and aimed to facilitate participation of persons who would otherwise not be able to participate in the study because of language barriers. Interviews lasted between 30 and 90 min (mean = 51.3 min, SD = 19.6).

### Data collection

**Figure 1.**
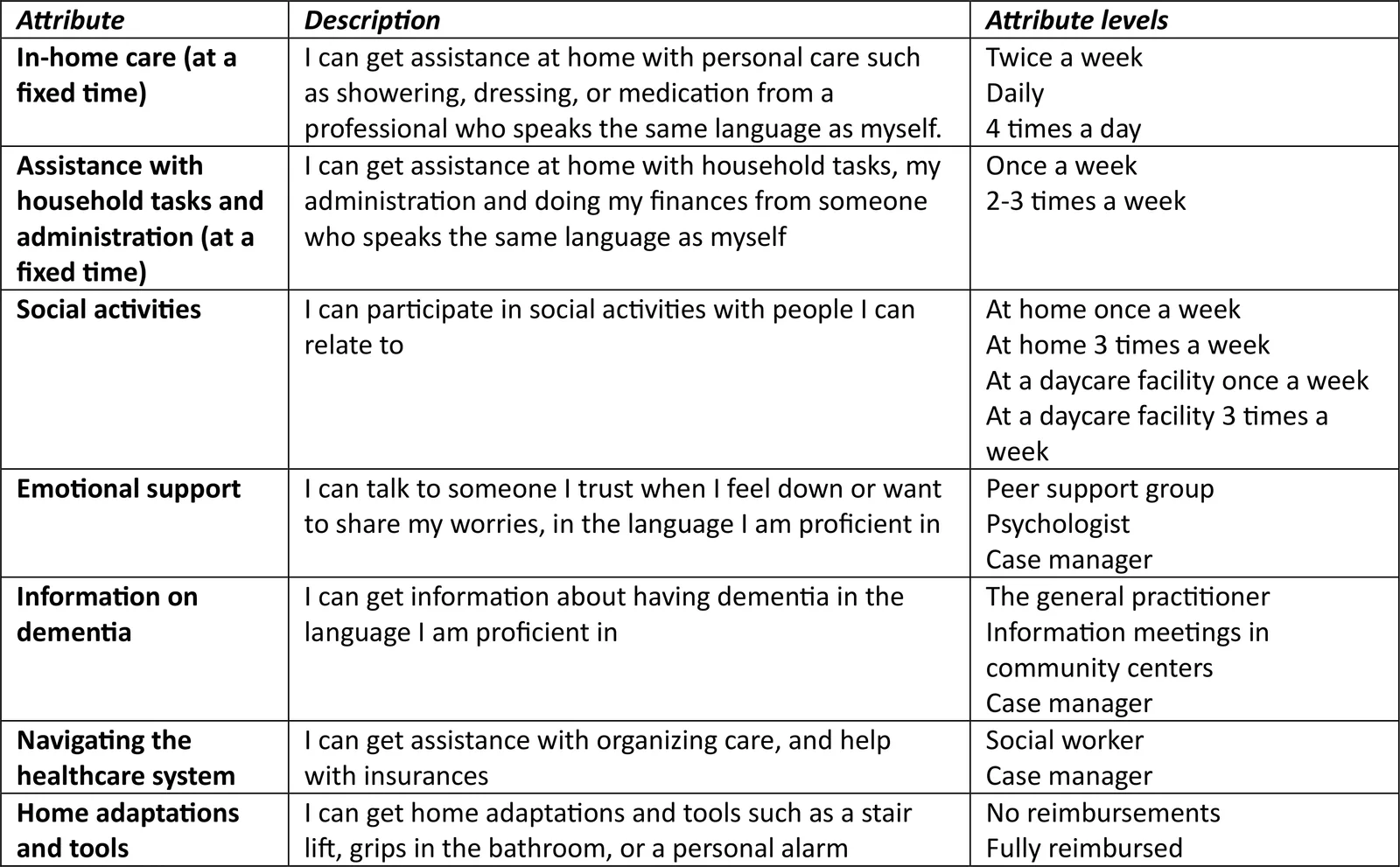
Attributes and levels used in the DCEs.

Participants completed eight DCE choice tasks, each presenting two hypothetical care packages defined by seven care attributes and their corresponding levels.^22,32^ Persons with dementia received a partial-profile design containing three to four attributes per task (Eight choice tasks in total) to reduce cognitive load, in accordance with evidence suggesting this improves feasibility.^25^ A full list of attributes and levels is provided in Figure 1, and an example choice task is presented in Supplemental Figure 2.

Interviewers actively facilitated the dialogue, which was crucial especially during dyadic rounds, to support persons with dementia in articulating preferences.^25^ Illustrations created by a professional graphic designer specializing in materials for cognitively impaired individuals were used to visually represent the attributes. The illustrations were then panel tested with clinicians and persons with dementia and informal caregivers to make sure they were appropriate. The DCEs were conducted using the Qualtrics platform,^33^ an online survey tool designed for user-friendly data collection. The sessions were audio recorded and transcribed verbatim in Dutch. In cases in which another language was spoken, a native speaker of that language transcribed the audio record directly in Dutch.

### Data analysis

Sociodemographic data included age, sex, educational background, migration background, whether there was a formal dementia diagnosis, and caregiver relationship. Cognitive functioning was assessed by using the Brief Cognitive Rating Scale (BCRS), a caregiver-reported measure that rates five domains of functioning (concentration, recent and past memory, orientation, and self-care) on a 7-point scale, with higher scores indicating greater impairment.^34^ Dementia severity was staged using the Global Deterioration Scale (GDS), which classifies individuals into seven levels ranging from no cognitive decline (Stage 1) to very severe decline (Stage 7).^35^ The Inclusion of Other in the Self (IOS) scale is a single-item pictorial measure in which participants choose one of seven overlapping circle diagrams to represent their perceived closeness to another person or group.^36^

Thematic analysis of the transcripts followed Braun and Clarke's six-phase approach: 1) familiarization with the data, 2) generation of initial codes, 3) search for themes, 4) review of themes, 5) definition and naming of themes, and 6) production of the final report.^37^ Deductive coding based on the DCE attributes was combined with inductive coding for new emerging themes. To enhance rigor, two transcripts were independently coded by a second researcher (IV), and coding was refined through group discussions with the research group (SEJ, BF, CG, JMV, IV). MAXQDA software was used to support data management and analysis.^38^

## Results

### Participant's characteristics

**Figure 2.**
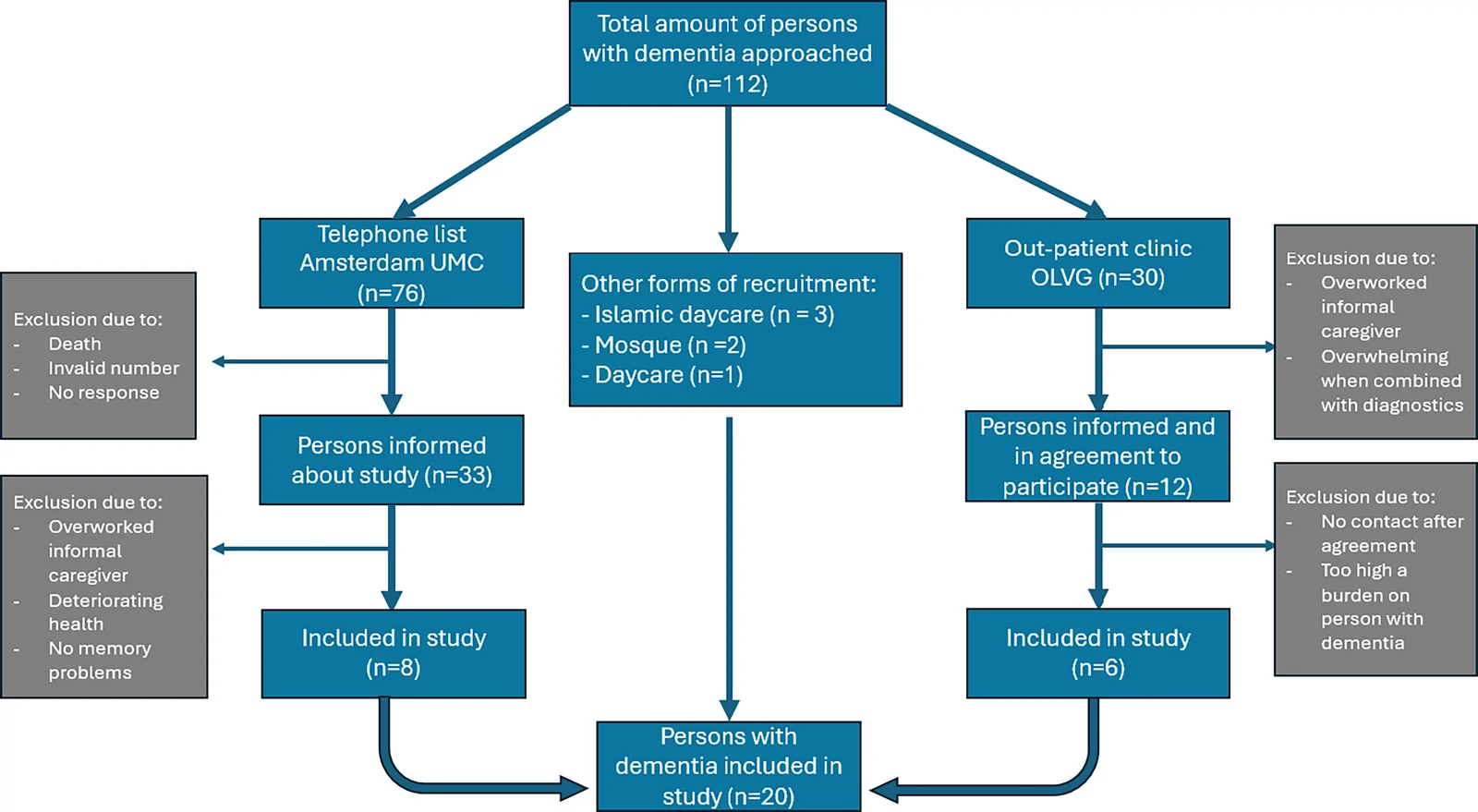
Flow diagram of the inclusion process over the course of 4 months (12/24–3/25).

In total, 112 dyads were approached for this study, leading to the recruitment and inclusion of 20 dyads (20 persons with dementia and 20 informal caregivers, total n = 40). Roughly half of the persons with dementia (50%) and informal caregivers (45%) had a migration background. Further details on the recruitment process and background characteristics are provided in Figure 1, 2 and Table 1.

**Table 1.** Participant characteristics.

	Person with dementia (*n* = 20)	Informal caregiver (*n* = 20)
Age (n,%)		
36–55		7 (35%)
56–65		4 (20%)
66–80	13 (65%)	6 (30%)
81+	7 (35%)	3 (15%)
Sex (n,%)		
Male	10 (50%)	6 (30%)
Female	10 (50%)	14 (70%)
Country of origin (n, %)		
The Netherlands	10 (50%)	11 (55%)
Morrocco	9 (45%)	7 (35%)
Pakistan	1 (5%)	1 (5%)
Sweden		1 (5%)
Living situation (n,%)		
Alone	4 (20%)	
With informal caregiver	11 (55%)	
With others	5 (25%)	
Relation to informal caregiver (n,%)		
Partner	9 (45%)	
Parent	10 (50%)	
Other	1 (5%)	
Form of education (n,%)		
No formal education	7 (35%)	
Primary school	3 (15%)	1 (5%)
High school	3 (15%)	4 (20%)
Secondary vocational education		2 (10%)
Higher professional education	3 (15%)	6 (30%)
Academic education	4 (20%)	7 (35%)
Formal dementia diagnosis (n,%)		
Yes	15 (75%)	
No	5 (25%)	
Global deterioration scale (GDS)-stadium (n, % of total participants)		
No cognitive decline (level 1)	1 (5%)	
Very mild cognitive decline (level 2)	3 (15%)	
Mild cognitive decline (level 3)	7 (35%)	
Moderate cognitive decline (level 4)	4 (20%)	
Moderately severe cognitive decline (level 5)	4 (20%)	
Severe cognitive decline (level 6)	1 (5%)	
Very severe cognitive decline (level 7)	0	
Inclusion of other in self (IOS)-scale, range 1–7 (mean, ±SD) *	6.35 (1.19)	5.95 (1.28)

*Higher scores on the IOS scale correlate to a feeling of being more connected to the other person.

### Main themes

Five main themes regarding AIP preferences were revealed: 1) burden of in-home care and care planning, 2) role and necessity of the case manager, 3) importance of accessibility and inclusivity of social activities, 4) need for trust and an established bond with healthcare providers and 5) prioritization of reimbursements of home adaptions and tools.

### Burden of in-home care and care planning

#### In-home care frequency

Persons with dementia often focused on in-home care intensity and sometimes needed prompting to consider other care aspects. Both groups generally preferred options with less in-home care, viewing it as less burdensome since it was sometimes seen as an invasion of privacy in the home. However, informal caregivers occasionally favored daily care for its continuity.

IC: “It is four times a day of course, that package […].”

*PwD: “Well, then I say, the less the better. So I would choose package one, even though I like the rest of that other package better. That's more important.”*

- *Dyadic interview*

#### Confrontation with the progression of dementia in the future

Although most informal caregivers had considered the idea of future in-home care, persons with dementia often stated that they had never thought about this topic in extensive detail. Both groups discussed the unpredictability of dementia. One participant with dementia experienced this unpredictability as emotionally distressing and requested to end the interview. While some participants found the topic overwhelming, others perceived it as challenging yet valuable, appreciating the opportunity to discuss and reflect on care planning.

*PwD: “I really don’t know how it's going to be.. So I find these all very scary things.. It all just takes on its own life in my head. So I just.. I just can’t say.. it's all too heavy for me to say. It's too soon.”*

- Interview with person with dementia

### The role and necessity of the case manager

#### Differences in familiarity with the case manager

In the individual interviews, persons with dementia were often unfamiliar with the case manager, frequently asking the interviewer for clarification on the term. If a case manager was involved, the informal caregiver helped them remember by providing context. The informal caregiver was often the main point of contact. Some mentioned a great connection with the case manager, while others had never heard of a case manager. Positive experiences with the case manager were tied to accessibility and good communication.

*PwD: “I don’t really know what to imagine when thinking of a case manager. Is that somebody that comes here or..?”*

*IC: “Yes, you know her. She comes here once every six weeks (…)”*

*PwD: “Oh her, yes she's nice, she can come if she wants.”*

- *Dyadic interview*

### The primary role of the case manager

In contrast, informal caregivers often prioritized the case manager when deciding between the two packages. Different roles of the case manager were discussed: emotional support, navigating the health care system, providing information and as a central contact person. It was often mentioned how the number of health care professionals involved could become burdensome. Even without positive experiences, both groups strongly preferred the case manager if it meant having them as central contact person. Lastly, the role of navigating the health care system was often preferred over emotional support by informal caregivers. Persons with dementia did not show a clear preference.

*IC: “Look, here you have one, two, three different people. And here you have everything by the case manager; the emotional support, the information and the navigating.”*

*PwD: “Central.”*

*IC: “Yes, because it's really sweet that everyone wants to come help you, but it's also burdensome to have so many people want things from you.”*

- *Dyadic interview*

### Importance of accessibility and inclusivity of social activities

#### Daycare frequency

Persons with dementia strongly prioritized social activities during the choice tasks but often left that three times a week was too frequent. When choosing between the daycare centers and social activities at home, most preferred the daycare centers, citing opportunities to meet new people. Informal caregivers often supported daycare, emphasizing the need for activities outside the home. Accessibility was important to both groups.

*IC: “No, we can go to the daycare every day, if that's what we’d want. We don’t do that because it would just be too much.”*

*PwD: “One time a week, it's only nice like that.”*

- *Dyadic interview*

### Need for culturally sensitive daycare

A clear distinction emerged between participants with and without a migration background. Those with a migration background emphasized the need for culturally sensitive daycare. Informal caregivers often stressed that the activities should align with the culture and/or religion of the person with dementia. Persons with dementia who experienced culturally sensitive daycare found comfort in various aspects of these activities.

*IC: “The day care is quite nice (..) We notice with older adults, that they still have a need to sit together and speak their own language (…) When surrounded only by Dutch people, they feel uncomfortable in terms of language, religion, and so on.”*

- *Dyadic interview, informal caregiver*

### Need for trust and an established bond with healthcare providers

Almost all participants considered emotional support when deciding between the packages. Preferences between the different options varied, but in every interview the need for trust and an established bond was emphasized. When a good relationship with the case manager had been established, participants would often prefer them over other sources of emotional support. The way emotional support should be provided, varied. Some participants mentioned the general practitioner, valuing small check-in moments during consultations. Participants with a migration background often stressed the difficulty of language barriers when communicating with healthcare providers.

*PwD: “I would prefer the general practitioner. I trust her. (…) Look you don’t go to her for this sort of stuff, but we have a good relationship, she always asks how I’m feeling.”*

- *Interview with person with dementia*

### Prioritization of reimbursements of home adaptations and tools

Most of the informal caregivers and persons with dementia strongly preferred full reimbursement for home adaptions and tools. Prioritization depended on their willingness and ability to pay out of pocket and expectations of needing significant home modifications. While some could not afford out of pocket costs, others felt they did not need the reimbursements or others would benefit more.

*PwD: “Well, let's pick this one. The world is already difficult enough. There are enough people who need this, I don’t.”*

- *Interview with person with dementia*

## Discussion

This DCE study, using a think-aloud protocol, explored and compared AIP care preferences of persons with dementia and their informal caregivers and provided in depth insight into their choice behavior. Five key themes emerged: (1) burden of in-home care and planning, (2) role of the case manager, (3) accessibility of social activities, (4) trust in healthcare providers, and (5) reimbursement of home adaptations and tools. Persons with dementia prioritized day-to-day aspects of care, such as in-home support and social engagement. Informal caregivers prioritized help with navigating the healthcare system, emphasizing the importance of the case manager as a central contact person. Both groups valued culturally inclusive services, trusted healthcare relationships, and full reimbursement for home adjustments.

Our findings align with previous research showing differences in care preferences between persons with dementia and their informal caregivers.^15,29^ Persons with dementia prioritized support that pertain to daily life, such as assistance with day-to-day activities, consistent with a prior study prioritizing care supporting AIP.^29^ However, we found that persons with dementia often found frequent care too intrusive and expressed emotional discomfort when reflecting on future care. This resonates with the findings of Sellars et al.^39^ and Piers et al.,^40^ who recognized that advanced care planning can be emotionally taxing and avoided due to unfamiliarity with dementia.

Informal caregivers in our study recognized the importance of in-home care, but nevertheless prioritized case management as a single point of contact to navigate the healthcare system. This diverges from the findings of Kampanellou et al.,^41^ who reported that informal caregivers considered in-home care to be one of the most important components of a home “support” package, possibly due to differences of dementia severity of participants in both studies. Our study participants were mostly in early to mid-stages (GDS 3–4), whereas Kampanellou et al.^41^ focused on later stages. Our finding emphasizes informal caregivers’ need for the involvement of a case manager in relation to navigating the healthcare system, reflects their position as the primary coordinator of care.^42^ This is supported in another study that emphasizes informal caregivers’ need for extensive support in organizing and managing care and caregiving responsibilities by.^43^ Even though persons with dementia were often unfamiliar with the concept of case management, they valued the idea of a central contact point such as a case manager, like informal caregivers. This strong preference among both groups became evident in the think-aloud part of the DCE. In at least 3 attributes of the choice tasks, the case manager was presented as a level, facilitating a dialogue between the person with dementia and informal caregiver about the potential role of a case manager. Notably, even participants with limited or no direct experience with a case manager expressed a desire for such a central figure. This aligns with a review identifying inconsistencies and gaps in care coordination roles as challenges in current healthcare system navigation.^42^

Both informal caregivers and persons with dementia emphasized the need for social activities, most preferring daycare over in-home options. This importance of social activities reflects earlier findings showing that persons with dementia highly valued opportunities for social and recreational activities.^15^ A recent study highlighted lack of access to meaningful social activities as one of the most frequently reported unmet needs among persons living with dementia.^44^ Similarly, Teahan et al.^45^ found that informal caregivers valued social activities as a means to address both the needs of the person they cared for but also their own, for instance by alleviating isolation and providing respite. Moreover, our study highlights the importance of (culturally inclusive) daycare for persons with a migration background. This finding is particularly relevant in light of a recent review of European dementia care guidelines, which revealed that existing recommendations rarely address cultural inclusivity or the adaptation of care to diverse populations.^46^ This reinforces the critical importance of integrating accessible and culturally inclusive social activities into dementia guidelines and care models, as their absence not only limits quality of life but also increases feelings of isolation and unmet care needs.

Finally, most participants had an outspoken preference for full reimbursements for home adaptations and tools. Even though this was the preferred option by most, by no means did all participants base their ultimate decision on this attribute. The preference for reimbursement often depended on participants’ financial means and their perceived likelihood of needing such adaptations. This relationship between ability to pay and willingness to pay is echoed in findings from a mixed methods study by Meijer et al.,^47^ who found a correlation between willingness to pay for care and income, age and education. Beyond individual circumstances, high out-of-pocket costs and unclear reimbursement pathways have also been highlighted as important ethical concerns and barriers to equitable access in dementia care.^48^ A systematic review^48^ on assistive technologies further emphasizes that such financial and structural challenges hinder implementation and increase the risk of inequalities in care.

A key strength of this study is the use of a DCE in combination with a think-aloud protocol. However, rather than focusing on quantitative DCE outcomes, the DCE functioned as an instrument to prompt participants to reflect on their preferences. This approach provided rich qualitative insights into how and why persons with dementia and their informal caregivers prioritize care services. Although recommended in DCE guidelines, this combination is rarely applied in dementia research.^49^ To our knowledge, no prior study has explored both the preferred form and level of care from a dyadic perspective. The findings of this study align with—and extend the preliminary qualitative interviews and focus groups conducted during the development of this DCE.^26^ While the preparatory work was essential for defining relevant attributes, the current study avoids duplication by transitioning from exploration to the quantification of preferences, examining how participants make specific trade-offs between those attributes. The think-aloud method also helped uncover unfamiliar care options, enabling interviewers to offer relevant information and support when requested. Beyond attribute development, we found significant added value in using the think-aloud technique during the final DCE design for three reasons. Methodologically, it allowed us to verify that persons with cognitive impairment could navigate the digital task and were making deliberate choices rather than guessing. Furthermore, since advanced care planning is often avoided in practice, the protocol revealed underlying assumptions that family members held about each other's preferences, helping to tease out the nuances of the dyadic relationship. Additionally, our oversampling of persons with a migration background is of great value in giving voice to a population that is generally underrepresented in research.^17,50^ Especially given their higher risk of getting dementia in comparison with the general population, their participation is crucial for accurate representation of the population with dementia living in the Netherlands.^16^

However, this study has some limitations. Persons from other backgrounds such as Turkish and Surinamese communities, who also have a higher prevalence of dementia,^16^ were not equally represented, which may limit the broader generalizability of the findings. However, another specific limitation Is that informal caregivers were not interviewed separately from the person with dementia. While this approach was chosen to minimize participant burden, it may have constrained informal caregivers from fully expressing their personal concerns. Recruitment primarily occurred through hospitals, meaning participants were already engaged with healthcare services, potentially introducing selection bias. Given that dementia diagnoses are often delayed, particularly in migrant populations, individuals with less access to healthcare may have been unintentionally excluded. Additionally, the recruitment rate was low (18%), largely due to the perceived burden by persons with dementia or their informal caregivers. Language barriers also posed challenges, as the DCEs were conducted in Dutch. While multilingual researchers facilitated communication by reading and interpreting tasks aloud, this may have introduced minor translation variability, especially with culturally or contextually complex terms such as “case manager”. Despite these challenges, the think-aloud method proved highly valuable, offering deeper insight into participants’ decision-making processes and revealing unfamiliar care concepts. We recommend incorporating think-aloud protocols in future DCE studies, especially with cognitively impaired populations, as they enrich data quality and support more informed participation.

The finding that persons living with dementia and their informal caregivers have differing care preferences has significant implications for research, policy, and practice. For research, it highlights the need to explore the reasons behind these differences, ensuring both groups are equally represented in studies, and refine methodologies like DCEs combined with think aloud protocols to capture diverse perspectives. Future research should expand on this design with larger and diverse samples to strengthen the evidence base and explore underlying reasons for these differences. For policy, this highlights the need to design inclusive care models that balance differing needs and allocate resources to support both groups effectively. It also underscores the importance of addressing barriers faced by marginalized populations, such as persons with a migration background, in accessing essential services like case managers and culturally sensitive daycare facilities. In practice, healthcare providers must develop person-centered care plans that integrate both individual and shared priorities. Our preliminary findings suggest that participants placed greater emphasis on case managers supporting families in navigating the healthcare system and providing information about dementia, while emotional support was perceived as a lower priority. This study directly addresses the evolving scope of dementia research by demonstrating the feasibility of DCEs within the cognitively impaired population. While DCEs are increasingly used to capture patient preferences in general healthcare, clear methodological guidance for their application in Alzheimer's disease and related dementias remains in its preliminary stages. By refining the “think-aloud” dyadic protocol, particularly among high-risk and underrepresented groups such as persons with a migration background, this work provides a framework for more inclusive and accurate preference elicitation. Such advancements are essential for the Journal of Alzheimer's Disease community, as they ensure that the “voice” of the person with dementia remains central to the design of future care models and policy reforms. By aligning research, policy, and practice with these insights, care models can be tailored to improve outcomes and enhance the quality of life for both persons with dementia and their caregivers. Continuation of this study is crucial to assess the feasibility of the study design with a larger sample size, and to provide stronger, quantifiable evidence regarding care preferences. Given the shift toward informal care in the Netherlands following the 2015 reforms, building a national evidence base on these needs is essential to inform policy and ensure both persons with dementia and their caregivers, with and without migration backgrounds, are represented in care planning and resource allocation.

## Supplemental Material

sj-docx-1-alz-10.1177_13872877261469138 - Supplemental material for Aging in place preferences of older adults with cognitive impairment and informal caregivers: A qualitative analysis of a discrete choice experiment Supplemental material, sj-docx-1-alz-10.1177_13872877261469138 for Aging in place preferences of older adults with cognitive impairment and informal caregivers: A qualitative analysis of a discrete choice experiment by Soukaina El Jaouhari, Caroline Geetan, Bachar Farousi, Luis I. Pilli, David Neal, Thomas Engelsma, Isabelle Vullings, Özgül Uysal-Bozkir, Floor Van Deudekom, Majon Muller and Janet Macneil Vroomen in Journal of Alzheimer's Disease
