# Supplementary material for: Aging in place preferences of older adults with cognitive impairment and informal caregivers: A qualitative analysis of a discrete choice experiment
Source: J Alzheimers Dis. 2026 Jul 23;113(1):366–76. doi: 10.1177/13872877261469138 (PMC13499922; doi:10.1177/13872877261469138)
Supplement: Supplementary file 1 [file sj-docx-1-alz-10.1177_13872877261469138.docx]

**Supplemental Material**

**Aging in place preferences of older adults with cognitive impairment and informal caregivers: A qualitative analysis of a discrete choice experiment**

**Supplemental Figure 1.** Description of study design used to create Discrete Choice Experiment.^1^

**Reference**

1.	Vullings I, Wammes J, Uysal-Bozkir Ö, et al. Eliciting preferences of persons with dementia and informal caregivers to support ageing in place in the Netherlands: a protocol for a discrete choice experiment. *BMJ Open* 2023; 13: e075671.

**Supplemental Table 1.** COREQ (COnsolidated criteria for REporting Qualitative research) Checklist

| **Topic** | **Item No.** | **Guide Questions/Description** | **Reported on**  **Page No.** |
| --- | --- | --- | --- |
| **Domain 1: Research team and reﬂexivity** | | | |
| *Personal characteristics* | | | |
| Interviewer/facilitator | 1 | Which author/s conducted the interview or focus group? | 8 |
| Credentials | 2 | What were the researcher’s credentials? e.g., PhD, MD | 8 |
| Occupation | 3 | What was their occupation at the time of the study? | 8 |
| Gender | 4 | Was the researcher male or female? | n/a |
| Experience and training | 5 | What experience or training did the researcher have? | 8 |
| *Relationship with participants* | | | |
| Relationship established | 6 | Was a relationship established prior to study commencement? | 7 |
| Participant knowledge of the interviewer | 7 | What did the participants know about the researcher? e.g., personal goals, reasons for doing the research | 7 |
| Interviewer characteristics | 8 | What characteristics were reported about the inter viewer/facilitator?  e.g., Bias, assumptions, reasons and interests in the research topic | 7 |
| **Domain 2: Study design** | | | |
| *Theoretical framework* | | | |
| Methodological orientation and Theory | 9 | What methodological orientation was stated to underpin the study? e.g., grounded theory, discourse analysis, ethnography, phenomenology,  content analysis | 7 |
| Audio/visual recording | 19 | Did the research use audio or visual recording to collect the data? | 8 |
| Field notes | 20 | Were ﬁeld notes made during and/or after the interview or focus group? | 8 |
| Duration | 21 | What was the duration of the inter views or focus group? | 8 |
| Data saturation | 22 | Was data saturation discussed? | n/a |
| Transcripts returned | 23 | Were transcripts returned to participants for comment and/or | n/a |
| **Domain 3: analysis and**  **ﬁndings** |  |  |  |
| *Data analysis* |  |  |  |
| Number of data coders | 24 | How many data coders coded the data? | 10 |
| Description of the coding  tree | 25 | Did authors provide a description of the coding tree? | n/a |
| Derivation of themes | 26 | Were themes identiﬁed in advance or derived from the data? | 10 |
| Software | 27 | What software, if applicable, was used to manage the data? | 10 |
| Participant checking | 28 | Did participants provide feedback on the ﬁndings? | n/a |
| *Reporting* |  |  |  |
| Quotations presented | 29 | Were participant quotations presented to illustrate the themes/ﬁndings?  Was each quotation identiﬁed? e.g. participant number | 10-13 |
| Data and ﬁndings consistent | 30 | Was there consistency between the data presented and the ﬁndings? | 14-16 |
| Clarity of major themes | 31 | Were major themes clearly presented in the ﬁndings? | 10-13 |
| Clarity of minor themes | 32 | Is there a description of diverse cases or discussion of minor themes? | n/a |

**Supplemental Figure 2.** Example of choice tasks (full and partial profile)

*Full profile*

*Partial profile*
